# Interactions of the Calcite {10.4} Surface with Organic Compounds: Structure and Behaviour at Mineral – Organic Interfaces

**DOI:** 10.1038/s41598-017-06977-4

**Published:** 2017-08-08

**Authors:** S. S. Hakim, M. H. M. Olsson, H. O. Sørensen, N. Bovet, J. Bohr, R. Feidenhans’l, S. L. S. Stipp

**Affiliations:** 1 Nano-Science Center, Department of Chemistry, University of Copenhagen, Universitetsparken 5, 2100 Copenhagen, Denmark; 2 DTU Nanotech, Department of Micro- and Nanotechnology, Technical University of Denmark, Ørsteds Plads, 2800 Kgs. Lyngby, Denmark; 3 Niels Bohr Institute, University of Copenhagen, Blegdamsvej 17, 2100 Copenhagen, Denmark

## Abstract

The structure and the strength of organic compound adsorption on mineral surfaces are of interest for a number of industrial and environmental applications, oil recovery, CO_2_ storage and contamination remediation. Biomineralised calcite plays an essential role in the function of many organisms that control crystal growth with organic macromolecules. Carbonate rocks, composed almost exclusively of calcite, host drinking water aquifers and oil reservoirs. In this study, we examined the ordering behaviour of several organic compounds and the thickness of the adsorbed layers formed on calcite {10.4} surfaces. We used X-ray reflectivity (XRR) to study calcite {10.4} surfaces that were prepared in three alcohols: methanol, isopropanol and pentanol and one carboxylic acid: octanoic acid. All molecules adsorbed in self-assembled layers, where thickness depended on the density and the length of the molecule. For methanol and isopropanol, molecular dynamic simulations (MD) provided complementary information, which allowed us to develop a surface model. Branching in isopropanol induced slightly less ordering because of the additional degree of freedom. Pentanol and octanoic acid adsorbed as single monolayers. The results of this work indicate that adhered organic compounds from the surrounding environment can affect the surface behaviour, depending on properties of the organic compound.

## Introduction

Calcite (CaCO_3_) is an important biomineral for organisms such as sea urchins, oysters and coccolithophores, a group of planktonic marine algae. Crystal growth is controlled by the presence of organic macromolecules^1–5^. Limestone and chalk, which form vast beds of sediments, serving as aquifers for water supply and reservoirs for oil and gas, are often composed of 95% or more biogenic calcite. Chalk is composed predominantly of coccoliths, which are made of 20 to 60 individual calcite crystals that are less than 1 μm in dimension. Their formation is controlled by the activity of complex polysaccharides^6–11^. In studies by Henriksen *et al*.^8^ using atomic force microscopy (AFM) and Yang *et al*.^9^ using molecular dynamics (MD) modelling, it has been shown that the interaction of complex polysaccharides with the calcite surface occur through hydroxyl and carboxylate functional groups. In oil reservoirs, the adhesion of hydrocarbon molecules, which contain a range of functional groups, controls the extent of oil release^12–14^. In carbonate rock aquifers, remediation of organic compound contamination is often limited by the affinity of the pesticides, chlorinated solvents or hydrocarbons to the calcite surfaces but for all of these systems, understanding about how different organic compounds interact with calcite is still lacking. Therefore our aim was to study how a set of simple organic molecules structure themselves on calcite, at the molecular level.

X-ray reflectivity (XRR) is particularly well suited for investigating the characteristics of solid-fluid and fluid-fluid interfaces and thin layers on flat substrates^15,16^. The technique probes the electron density profile at interfaces, revealing information about surface roughness and atomic structure^17–23^. It is particularly useful on minerals such as calcite, which can be cleaved to provide surfaces that are remarkably flat^20,24–29^. There are studies of self-assembled monolayers of organic compounds on e.g. Si and SiO_2_ surfaces, performed using XRR^30,31^. Supplementary information derived from MD simulations is useful for confirming interpretations of XRR data, such as derived electron density profiles^32–35^. We have used XRR to investigate the interaction of four simple molecules, methanol, propanol, pentanol and octanoic acid on freshly cleaved calcite {10.4} surfaces. Previous studies using AFM, XRR and MD simulations^34,36^, have shown that ethanol forms ordered layers on calcite surfaces. A recent study by Bovet *et al*.^35^, using X-ray photoelectron spectroscopy (XPS) and MD simulations, showed a pattern in the coverage of low molecular weight alcohols.

Calcite has a rhombohedral crystal structure, producing perfect cleavage faces. The alternating Ca^2+^ and CO_3_^2−^ on calcite {10.4} generate a polar, hydrophilic surface. Water is the simplest molecule with hydroxyl (-OH) functional group and it interacts strongly with calcite both by electrostatic interaction, between Ca_surface_ and O_hydroxyl_ and by hydrogen bonding between O_surface_ and H_hydroxyl_^24–28,37–40^. Bohr *et al*.^28^ have shown that the thickness of the water layer adsorbed on freshly cleaved calcite surface is unaffected by the relative humidity. The low molecular weight alcohols have both a hydrophobic, methyl group (-CH_3_) and a hydrophilic, hydroxyl group (-OH). Thus they can serve as models for more complex organic compounds that are soluble in aqueous solutions and that are able to interact with mineral surfaces. Clean, freshly cleaved calcite surfaces are hydrophilic but a single monolayer of organic molecules can change their behaviour from hydrophilic to hydrophobic^33–37^. Tidswell *et al*.^41^ have previously studied the wetting transition of silicon wafers Si/SiO_2_ and silicon wafers treated with CH_3_ terminated alkylsiloxane/SiO_2_/Si in the presence of cyclohexane and methanol using the XRR and undertook a comparison with results from the contact angle measurements interpreted in the framework of the Hamaker constant. They implemented a method that utilizes the temperature gradient to investigate the wetting transition. A comprehensive review of the wetting phenomenon can be found in a study by Bonn and Ross^42^ where the wetting transition from a prewetting layer with a thickness on a molecular length scale takes places to a macroscopically thick layer. For example an interesting study reveals the effect of various degree of deuteration of methanol in the binary liquid system cyclohexane-methanol^43^.

The alcohols adsorb to hydrophilic calcite through their polar, -OH end, while their hydrophobic end forms a new hydrophobic surface. Studies by Sand *et al*.^34^ and Cooke *et al*.^44^, using a combination of AFM and MD simulations, showed that ethanol attaches more strongly on calcite surface than water, forming an ordered and stable adsorption layer. An MD study by Keller *et al*.^33^ demonstrated that ethanol replaces water at the surface if the liquid is a water/ethanol mixture. A recent study by Ataman *et al*.^45^ used density functional theory (DFT) and XPS to show that carboxylic acids (R-COOH) adsorb more strongly than alcohols (R-OH) and water (H-OH) and these interact more strongly than aldehyde (R-CHO). They reported that side groups of organic molecules influence the adsorption behaviour of the hydroxyl functional group.

XRR has been used in several studies to observe the interaction of water on calcite^24–28^. Pasarin *et al*.^36^ investigated ethanol adsorption on calcite but we are not aware of any XRR studies of larger organic compounds. Organic molecules with the same functional groups have different properties as a result of their structure and their side groups^45^ so additional information about their structuring at the surface would improve our understanding. Some recent MD studies^33,34,36,44^, that have primarily focused on interactions between straight chain organic molecules and calcite, have frequently used ethanol as a model for organic molecules with hydroxyl groups, including quite complex polysaccharides. The purpose of the present study was to further investigate surface ordering of other organic molecules on calcite surfaces for four different systems with varying molecule structure but under common condition (ambient condition). One of our main interests was to gain a deeper understanding about the interaction of branched molecules with calcite so we chose isopropanol, which has similar molecular size and bulk liquid density as ethanol. The other aim in this study was to investigate the thickness of the adsorbed layer based on molecule length and density. So we chose pentanol and octanoic acid, which have longer chains and higher bulk density. Carboxylic acids are common in natural systems, as humic and fulvic acids in groundwater, rivers, lakes and the ocean and they are common polar components in crude oil. Because of their very polar functional groups, molecules with carboxyl groups act as surface active agents.

## Results and Discussion

### Small Molecule Surface Films - Methanol and Isopropanol

**Figure 1:**
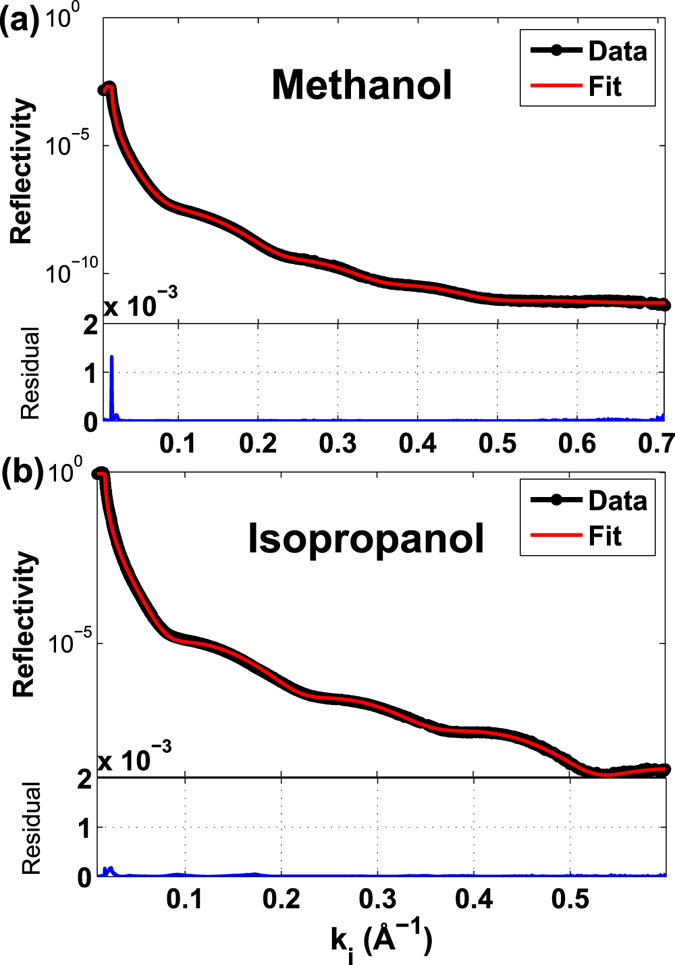
The best fit surface models. The best surface model (red line) fitted to the reflectivity data (black dots) of (**a**) methanol and (**b**) isopropanol adsorbed on calcite {10.4}. Residuals for the fit are plotted below each plot.

Reflectivity data for methanol and isopropanol are presented in Fig. 1a and b, where we depict the best fit surface models. The measured reflectivity intensity (black dots), presented in Fig. 1, was used to fit the surface model (red line) and is plotted as a function of the perpendicular component, k_i_ of the incoming k vector, i.e. k_i_ = (Q/2) = (2π/λ) * sin(θ), where Q represents the scattering vector (Å^−1^) and λ represents the wavelength (Å). A χ^2^ based R-value is calculated from the residuals, which are plotted at the bottom of the reflectivity curves, showing the goodness of the fit.

**Figure 2:**
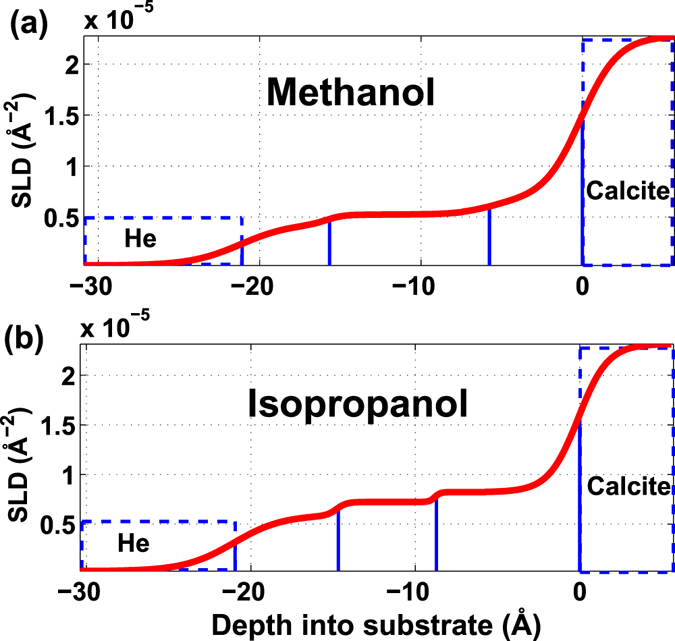
Scattering length density (SLD) based on the fitted model. Calculated SLD for (**a**) methanol, with total thickness of 21 Å and (**b**) isopropanol, with a total thickness of 20.1 Å.

Figure 2 shows the calculated scattering length density (SLD) as a function of depth into the calcite substrate. The best fit models suggest a 21 Å thick layer of methanol and a 20 Å thick layer of isopropanol adsorbed on the calcite surface (Fig. 2a and b). The results of the fit are compiled in Table 1. In the estimations for the theoretical molecule lengths, in Table 1, the thickness of the first molecular layer of the organic compound on the calcite surface is estimated to be the distance from the Ca ion in the calcite surface to the end of the molecule, i.e. the distance from Ca to the O ion in the hydroxyl group, which is 2.07 Å for alcohols (Fig. 4) and 2.32 Å for carboxylic acids, plus the distance to the end of organic molecule^45,46^. The results are in good agreement with the previous study by Pasarin *et al*.^36^, who showed that ethanol forms ordered layers on calcite with a total thickness of 20.3 Å, and is composed of two layers with thicknesses of 5.4 Å and 14 Å for the first and following layer (Theoretical length of the ethanol molecule is 6.0 Å including distance to the surface). The roughness of the calcite surface in that study was 0.7, and the fitted model suggested a density of 0.8 g/cm^3^ for the ordered ethanol layers. The bulk density and layer thickness for isopropanol and methanol (Table 1), are similar to those of ethanol, and considering the similarity of the molecular structures, we expect similar attachment, through -OH functional groups, and similar intermolecular and lateral interactions within the bulk layer. All three compounds developed an adsorbed layer with a total thickness that corresponds to at least three molecular layers of alcohol. The decreasing trend in the alcohol density in the upper layers (alcohol-2 and alcohol-3) is indicating that these layers are less ordered compared with the first layer (alcohol-1).

**Table 1:**
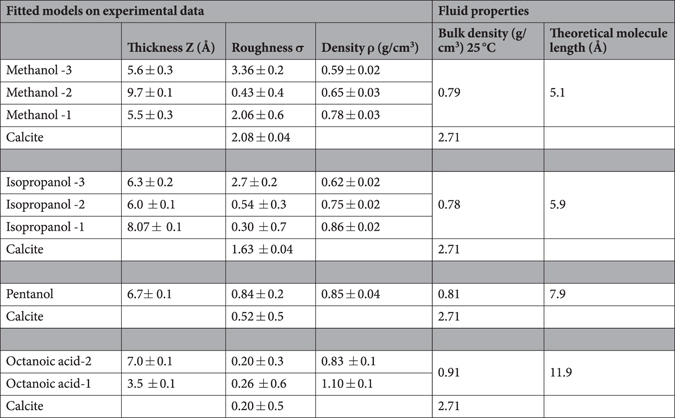
The tabulated parameters are obtained from fitting the reflectivity data with box models. For models with more than one layer, the layer closest to the calcite surface is indexed 1 (e.g. Methanol-1). The density for calcite (2.71 g/cm^3^) and helium (0.03 g/cm^3^) were kept fixed during the fitting procedure, and their thicknesses were infinite. Error estimates were obtained for every parameter by varying the parameter until the χ based R-value is changed by 5%^70,71^. The method does not reveal correlations between the parameters. On the right side, theoretical bulk density and the molecular length of the molecules are given for comparison.

### Modelling results

To further interpret our reflectivity data and better understand the alcohol-calcite interaction, we complemented the experimental data with MD simulations. We were interested in the effect of the molecule size and structure of the organic compounds and we wanted to relate our results with the previous studies of ethanol^36^. We simulated n-propanol and isopropanol and extracted the density profiles. The two propanol isomers have similar bulk liquid densities to ethanol. N-propanol has a straight chain structure whereas isopropanol is branched.

**Figure 3:**
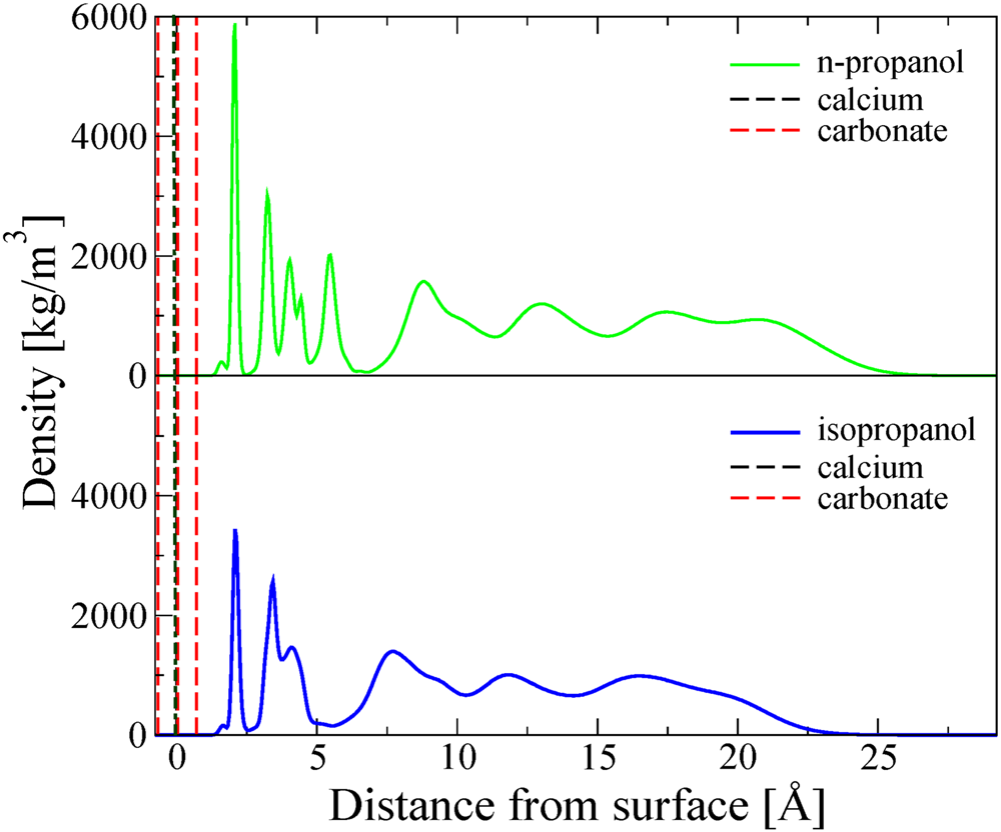
Mass density predicted for n-propanol and isopropanol on calcite {10.4}. The distances are recorded with reference to the calcite plane, defined by calcium, carbon and one of the oxygen atoms (black dashed line). For each carbonate ion, a second oxygen is above this plane and third is below (red dashed lines).

In Fig. 3, we present the nuclear density obtained from MD simulations. Both propanol isomers form thin films on the calcite slab, as we also found in our isopropanol experiment. These films were not uniform in density perpendicular to the calcite surface but formed distinct layers with propanol enrichment and depletion, which suggested that the molecules ordered themselves into a structured density profile. This is consistent with results from ethanol studies^33,34^. The molecular layers closest to the surface (1.3 to 6.6 Å for n-propanol and 1.3 to 5.4 Å for isopropanol) were well ordered, which can be seen from the sharp, well defined density peaks, and the molecules adsorbed in a close packed form. This is consistent with XPS results from calcite with adsorbed methanol, ethanol and propanol^35^.

The most intense peak for both alcohols (Fig. 3) is from the hydroxyl oxygen at a distance of 2.07 Å perpendicular to the surface. In bulk calcite, Ca is coordinated with O of the carbonate ion, so at the termination of the bulk structure, where the balance of forces between atoms is lost, Ca would attract O from ethanol to complete its coordination. The next intense peaks represent the CH_n_ groups. This peak structure reveals that the first layer of molecules adsorbs perpendicular to the calcite surface and that it has significant interaction with it.

**Figure 4:**
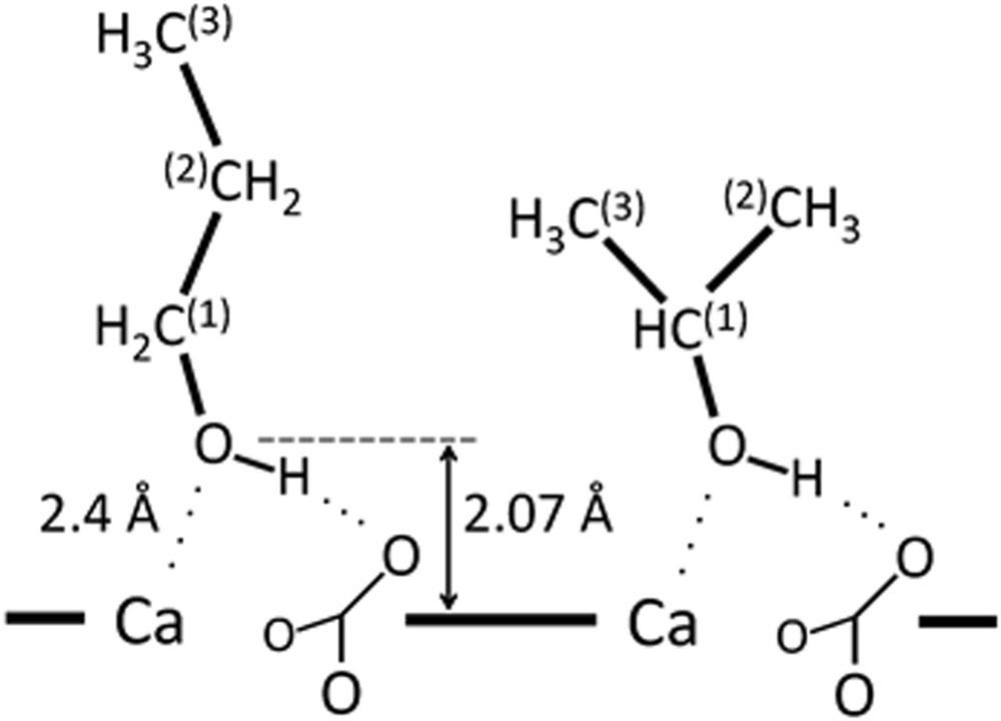
Coordination to the calcite surface for the two propanol isomers. Propanol forms strong interactions through hydrogen bonding and electrostatic interactions between O_alcohol_ and Ca_surface_. The carbon superscript denotes the atom numbering used in the next paragraphs.

The coordination of two propanol isomers to the calcite surface is shown in Fig. 4. Both alcohols attach through hydrogen bonding from the OH group of the alcohol to one of the carbonate oxygen atoms and through electrostatic interactions between the hydroxyl oxygen atom and the calcium ion. For n-propanol, the density contributions from Carbon Atoms 1, 2 and 3 are visible at distances 3.23, 4.03 and 5.46 Å from the surface (Figs 3 and 4). For isopropanol, the carbon dominated peaks are split in two: the peak representing Carbon Atom 1 is 3.42 Å from the surface and the contribution from the two identical carbon atoms, labelled 2 and 3, is 4.10 Å from the surface (Figs 3 and 4). The density peak for these atoms is significantly broader, which is consistent with a larger flexibility in orientation at the surface for isopropanol than n-propanol. Because both propanol isomers have the same functional group attached to the calcite surface, and Carbon Atom 1 is covalently linked to the hydroxyl group, the two first peaks are more or less identical in the density profile whereas the remaining peaks are different for the two systems. The broadening in the third and following peaks shows that the remaining carbon positions are more flexible.

The second and following molecular layers were less ordered than the first and show an accumulation of relatively free molecules at distances 8.8, 13.0 and 17.5 Å from the surface for n-propanol and 7.7, 11.8 and 16.5 Å for isopropanol. As a consequence, there are corresponding depletion zones at distances 11.3, 15.3, and 19.4 Å from the surface for n-propanol and 10.3, 14.1, and 18.3 Å for isopropanol. These zones of higher and lower density are expected in an ordered structure, just as there are planes of higher and lower atomic density in crystalline solids, the precise spacing of these enrichment and depletion zones should be considered with caution because the nuclear density is centred at the atom positions of each molecule whereas the electron density is distributed over the entire molecule. These layers are to a certain degree governed by polar surface-alcohol interactions but originate more likely from oscillatory forces^47^.

The simulated density profiles in this study are consistent with the results for ethanol on calcite, presented by Pasarin *et al*.^36^ and Bovet *et al*.^35^. They reported a thickness of 5.4 Å for the first adsorbed layer followed by a narrow density depletion gap (0.9 Å) and a more disordered ethanol layer (14 Å). Our MD simulations also predicted a narrow nuclear density gap after the first surface layer of self-organized propanol, before the remaining propanol. This gap is found above 6.6 Å from the surface for n-propanol and above 5.5 Å for isopropanol. This is consistent with isopropanol and ethanol being about the same length and n-propanol being 1.1 Å longer.

Our XRR data do not reveal depletion in the SLD after the first molecular layer as observed by MD predictions. The gap, if it is present, is only marginally detectable in this current study, considering the sample roughness and the instrumental resolution. Another possibility for not observing the gap could be because trace impurities and other isomers are present. Finally, the lateral interaction is an important factor that also must be considered when it comes to methanol and isopropanol compared with ethanol. The way molecules pack on the mineral surface has an important influence on surface coverage and the lateral forces that influence layer ordering. Using heat of adsorption measurements, DFT and MD simulations, Okhrimenko *et al*.^48^, showed that on calcite, methanol interacts more weakly laterally than the other alcohols. Their experimental results also indicated that ethanol has higher adsorption affinity on calcite surface than isopropanol although DFT and MD simulations suggested equal bonding strength.

### Single Monolayers - Pentanol and Octanoic Acid

**Figure 5:**
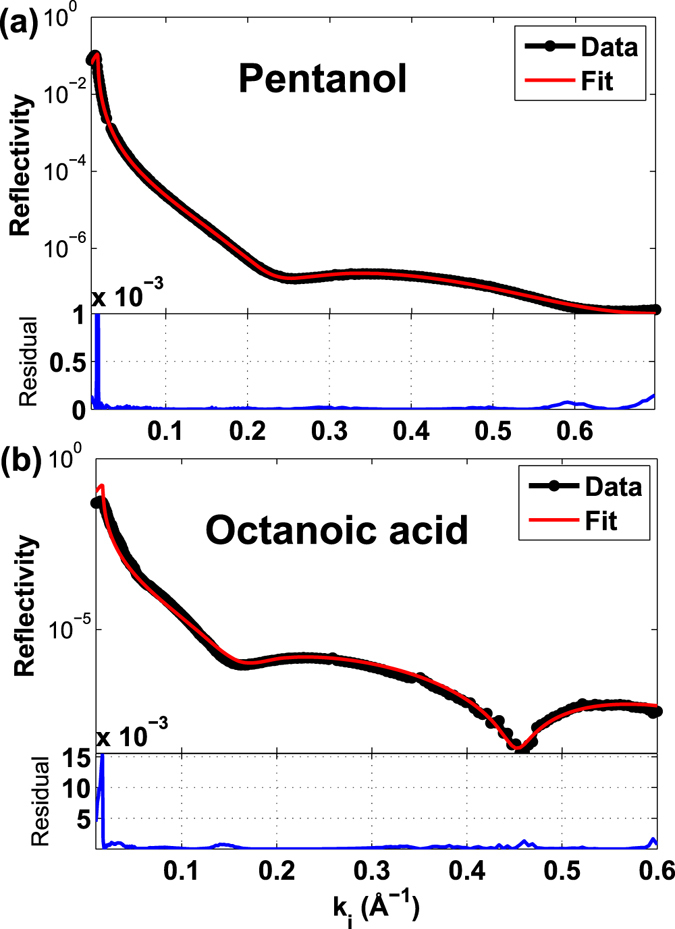
The best fit surface models. The best surface model (red line) fitted to the reflectivity data (black dots) of (**a**) pentanol and (**b**) octanoic acid on calcite {10.4}. Residuals for the fit are plotted below each plot.

**Figure 6:**
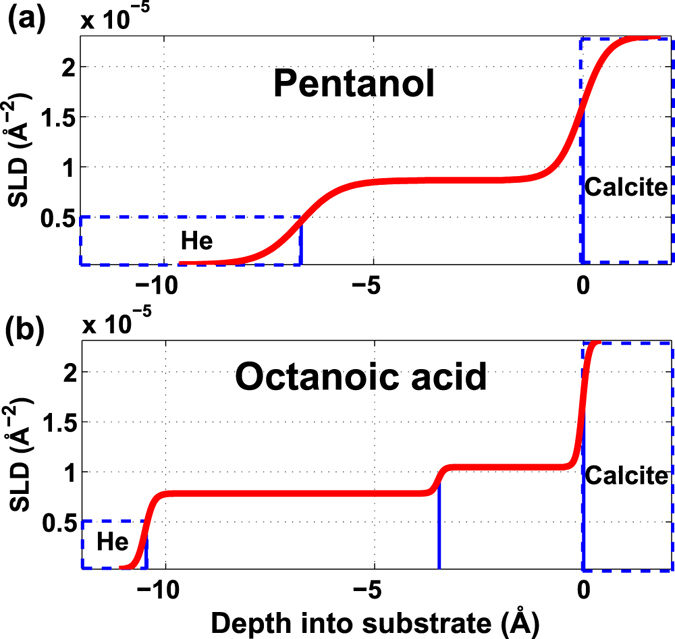
Scattering length density (SLD) based on the fitted model. Calculated SLD profile for (**a**) pentanol, with a total thickness of 6.7 Å and (**b**) octanoic acid, with a total thickness of 10.5 Å.

Reflectivity data for pentanol and octanoic acid were fitted using the same procedure as for the short chained molecules. The model that best represented the experimental data for pentanol was a single layer that reached 6.7 Å out from the calcite surface (Table 1 and Figs 5a and 6a). This can also be seen from a weak oscillation on the reflectivity curve in Fig. 5a. This thickness corresponds reasonably well with the length of a single pentanol molecule adsorbed on calcite, i.e. 7.9 Å (Table 1). The results agrees well with the study by Bovet *et al*.^35^. They have shown using MD simulations that pentanol interacts with calcite through the same hydroxyl functional group, by hydrogen bonding and electrostatic interaction, with its fatty tail standing away from the surface with molecule length of approximately 7.9 Å.

Pentanol molecule is longer than methanol, ethanol and isopropanol so there is more possibility for lateral interactions between the molecules in the first adsorbed layer and there is too little drive for the molecules in the subsequent layers to order themselves above the hydrophobic terminations of the first layer. This phenomenon may be a sufficient cause for adsorbed pentanol layer to be limited to one monolayer.

Octanoic acid also adsorbs to calcite with its polar functional group, while its fatty end stands away from the surface. The double bonded oxygen atoms of the carboxyl group (-COOH) interact electrostatically with surface calcium and the hydrogen interacts with the surface oxygen leading to hydrogen bonding^45,49^. For octanoic acid, a fit of the model to the reflectivity data indicated an adsorbed layer with a total thickness of 10.5 Å (Table 1 and Figs 5b and 6b). The single monolayer can clearly be seen from the single and wide oscillation on the reflectivity data in Fig. 5b. This is in agreement with the length of an octanoic acid molecule adsorbed on calcite, 11.9 Å (Table 1). The apparent difference may indicate that the tail of the molecules from the first carbon atom of the functional group are tilted at an angle, this is also observed for octanoic acid on ZnO (0001) surface in a DFT study by Islam *et al*.^50^. Although, molecular disorder could also contribute to the apparent thinning of the layer. This is due to the interaction of carboxyl groups with the polar surfaces and also the lateral interactions of the bounded carboxylic acids, which is discussed in details in a study by Ulman^51^. For the surface model, we fitted a two box model, where we split the density into the head group with a higher density and the alkyl tail with slightly lower density (Fig. 5b). In this model, the best fit model, the thickness of the first box for the head group (CH_2_-COOH), is 3.5 Å which corresponds very well to the distance from above the Ca atom of the surface to the second carbon atom of the acid, and the thickness of the second box, the rest of alkyl tail (-C_6_H_13_) is fitted to 7 Å, which can be explained by a tilting angle of *β* = ArcCos(7 Å/8.53 Å) ≈ 35°, where *β* is the angle between the tilted molecule chain and the normal to the surface, and calculated length for the tail (estimated distance from the second carbon atom to the last hydrogen cloud of the molecule) is ≈8.53 Å^46^. Furthermore, the ratio of absolute densities in each box ρ_box-1_/ρ_box-2_ = 1.1/0.83 = 1.34 is in approximate agreement with scattering electron densities in each box, [31(e)/3.5(Å)]/[49(e)/7(Å)] = 1.26. A one box model was also tested, but the fit was significantly poorer.

Similar to pentanol, octanoic acid has a long alkyl chain so the adsorbed layer is limited to one monolayer and ordering does not continue beyond the first layer.

The observed behaviour of pentanol and octanoic acid is relevant when considering the behaviour of mineral surfaces in nature and in industry, where natural solutions are a mixture of organic molecules, such as humic acids, polysaccharides, the hydrocarbons and complex polar molecules in crude oil, as well as all of the organic compounds in living systems where organisms create biominerals. For all four organic compounds on calcite, which we studied, we find a slightly higher density of the first adsorbed layer of the organic compounds. This shows that this layer is more ordered than the subsequent layers (Table 1).

The value of the roughness parameters decreases from 2.08 for methanol to 0.2 for octanoic acid. The density of calcite is about 2.7 g/cm^3^, which is nearly three times as large as the alcohol/carboxylic acid, the measured calcite roughness is therefore dominated by the morphology of the calcite surface, i.e. by steps, islands, etc. If we assume that these surface features are essentially as they appear from the cleavage process, then the roughness tells us about how successful the cleavage was to obtain a flat surface. In the present case cleaving calcite in pentanol and octanoic acid leads to significantly flatter surfaces than cleaving in methanol and isopropanol. Moreover, cleaving calcite in air results in even rougher surfaces. It is our experience that the cleavage in liquids generally results in better surfaces than dry cleavage. This is probably because of the liquid lubricates the interface between the scalpel blade and the crystal surface providing a smoother cutting procedure. This is an interesting phenomenon that needs to be further studied how these organic compounds with different bulk properties make bonds with the calcite surface at the atomic level during the process of cleavage, resulting in flatter cleavages.

The strong affinity of organic molecules for calcite, their ability to order themselves and attach strongly, is consistent with previous studies, showing all mineral surfaces such as calcite, quartz, feldspar, mica and clay are covered with at least one layer of organic material^14,37^. Self-assembled monolayers of long alcohols (higher than decanol) have previously also been observed on other surfaces than mineral surfaces. Haddad *et al*.^52^ have characterised octadecanol on Si(001) by XRR and studied the structure of the alcohol monolayer as function of temperature by following the thickness of the monolayer which gets slightly thinner when raising the temperature above the alcohol melting point, indicating that all molecules are still roughly aligned with surface-normal. Similarly the structuring of a Langmuir self-assembled monolayers of 1-decanol and 1-dodecanol on a water surface has been studied by Berge *et al*.^53^ using grazing incidence X-ray diffraction. Furthermore, Rieu *et al*.^54^, have studied the long chain alcohol monolayers at air-water interfaces by X-ray reflectivity.

## Conclusions

We investigated adsorbed layers of methanol, isopropanol, pentanol and octanoic acid on freshly cleaved calcite {10.4} surfaces that were cleaved in the organic compound of interest, using XRR and MD simulations. Methanol and isopropanol are comparatively similar in bulk density and molecule length, whereas pentanol and octanoic acid has a longer chain and slightly higher bulk density. All four molecules bind to the calcite surface through their functional group, while their ability to form ordered layers is influenced by the density and structure of the molecule chain.

The results from XRR and MD show that small organic molecules, namely methanol and propanol, organize themselves on the calcite {10.4} surface and form structures with both lateral and vertical order. Modelling predicts, as expected, that the organic molecules are bound to calcite {10.4} with their hydrophilic functional groups through strong interactions, standing perpendicular to the surface with their aliphatic tail pointing away. The smaller organic molecules, methanol and isopropanol, formed layers with similar total thickness (20 to 21 Å) and the densities determined for the adsorbed material is consistent with their bulk densities. The results agree well with the previous study by Pasarin *et al*.^36^, where ordered ethanol layers on calcite were found to be 20 Å thick. The two other organic compounds, pentanol and octanoic acid, with slightly longer molecule lengths and higher densities, also formed ordered adsorbed layers but the thickness was 6.7 Å for pentanol and 10.5 Å for octanoic acid. This is equivalent to a single molecular layer, including the molecule length plus the binding length to the calcite surface. For pentanol, this is 7.9 Å and for octanoic acid, it is 11.9 Å.

MD simulations allowed us to compare the behaviour of n-propanol, which has a straight chain structure, with isopropanol, which is branched. The density peak for the two carbon atoms that represent the branch of isopropanol in the first adsorbed layer is significantly broader, which is consistent with more flexibility in orientation and less dense structure for isopropanol than n-propanol.

Our results demonstrate that simple organic molecules form ordered adsorbed layers on calcite and the layer thickness is defined by their character. This implies that in nature and in industrial processes, where there is a multitude of possible organic adsorbates, no calcite surface is completely clean. Organic compounds from the surrounding environment would adhere to the surface and depending on their properties, could alter the surface properties and behaviour.

## Methods

### X-ray Reflectivity and Sample Preparation

Calcite crystals used in this study were cleaved just before the experiments from single crystals of optical quality Iceland spar. We cleaved the samples using the method described by Stipp and Hochella^37^. All tools and the calcite crystal bar were cleaned and rinsed in the organic compound before the crystal was merged in the organic liquid to be cleaved. Gentle pressure was applied repeatedly in parallel to the {10.4} surface, until the crystal cleaved, immersed in the organic compound, and the freshly cleaved surface is completely covered in the organic liquid. We used freshly cleaved surfaces, without any exposure to the air, to minimize adventitious carbon contamination, which could change the surface properties and behaviour. The quality of the fresh {10.4} surface was inspected by its reflection of visible light. The sample was kept in the liquid until it was mounted in the sample cell. The crystal, still covered by a thin layer of the liquid, was quickly and carefully moved from the liquid and placed in the cell. The cell was sealed and filled by the He/organic vapour. This procedure took less than a minute.

For all experiments, the atmosphere inside the cell was kept saturated with the organic compound by constantly bubbling helium gas through the organic liquid into the cell, such that the cell is kept at a constant elevated pressure of the He/organic vapour. The system was allowed to equilibrate for an hour while aligning the crystal in the X-ray beam. The assumption of a saturated gas phase was not independently verified. Because of the strong tendency for strong substrate binding and therefore for the formation of a solid layer which would introduce strong stepwise features in isotherms, we believe that possible lack of complete saturation would not drastically change the layer thickness. In a previous study by Bohr *et al*.^28^ for water layer on calcite the thickness of the adsorbed water layer was found to be constant in the range of 10–100% humidity.

Experiments with methanol, isopropanol and octanoic acid were conducted in the closed cell, covered with a cylindrical Kapton window, at the I811 beamline at the MAX IV Laboratory, Lund, Sweden, with beam energy of 12.4 keV. The experiment under pentanol was performed in a closed sample cell covered by a quartz glass dome at the I07 beamline at the Diamond Light Source, Didcot, UK, with beam energy of 18 keV. All organic compounds were of >99.5% purity (supplied by Sigma-Aldrich). XRR data was recorded on a 2D Pilatus 100 K detector up to the first Bragg peak. Adjusting the exposure time and beam filters for maximal signal-to-noise ratio at all angles. The XRR data were integrated using the method by Straasø *et al*.^55^. The integrated intensities were corrected for background and scaled according to the incoming beam intensity.

### XRR Data Fitting

Reflectivity intensity is proportional to the absolute square of the Fourier transform of scattering length density (SLD) in the vertical direction to the surface. Thus, obtaining the SLD structure from the measured reflectivity data leads to the well known phase problem^17,18^. This phase problem from 3-dimensional crystallography is overcome by the simpler task of making one dimensional models in an attempt to fit the experimental reflectivity data. Therefore, the density profile can be obtained from a best fit surface model. To fit a most probable surface model to the experimental data, we used a program developed in MATLAB, R2010b, originally implemented in a study by Whiting *et al*.^56^, implementing the Parratt method^17^ as outlined by Zhou *et al*.^57^. For details on reflectivity studies see Tolan and Press^58^ and Fermon *et al*.^59^. We tried a number of box models to fit the data for each organic compound, where each box or layer was represented by three parameters, thickness (Z), mass density (ρ) and roughness (σ) in a way that ensured consistency between the calcite-molecule systems. For all refinements there are at least two roughness parameters one for the solid-liquid interface and one for the liquid-gas interface. The roughness is assumed to be Gaussian. In all the models, we used two boxes to represent the inorganic layers, one for the calcite substrate and one for the helium atmosphere. The other boxes were used to model the adsorbed organic layer. To evaluate how well the fitted model represented the data, the software implements a χ^2^ based R-value, calculated from the residuals that are plotted below the fitted models in the figures.

### Molecular Dynamics Modeling

The simulation system was set up by creating a calcite slab with planar {10.4} surfaces, using Material Studio^60^. This face is the most stable and most common for calcite. Simulations were performed for systems of n-propanol and isopropanol. We added 500 alcohol molecules close to but not touching the surface. The system energy was then minimized with the GROMACS package^61–63^ using the steepest descent algorithm to attain a low energy configuration. Molecular dynamics simulations were performed with periodic boundary conditions, constant temperature to obtain an NVT canonical ensemble, using particle-mesh Ewald summation for long range interactions, an Andersen thermostat and the leapfrog algorithm^64,65^. VMD^66^ was used for visualization and analysis. The 6 layer calcite slab was 48.576 Å × 49.90 Å in *x* and *y* directions and the z direction was extended to 250 Å to avoid artificial interactions with image slabs resulting from the periodicity. The three bottom layers were frozen in their bulk positions to minimize artificial distortions that could arise from using a thin truncated slab rather than a nanometer scale crystal. The force field used in this study was a hybrid of the AMBER and Pavese potential functions, as previously used by Sand *et al*.^34^. The calcite parameters were adopted from Pavese *et al*.^32^ and the alcohol parameters came from AMBER^67,68^. The potential for the cross terms were those proposed by Freeman *et al*.^69^. Simulations were run at 300 K for more than 50 + 50 nanoseconds (ns), where the first 50 ns simulation was discarded as equilibration and the second 50 ns simulation was used to collect averaged adsorption properties.
