# Publisher Correction: Interactions of the Calcite {10.4} Surface with Organic Compounds: Structure and Behaviour at Mineral – Organic Interfaces

**DOI:** 10.1038/s41598-018-28935-4

**Published:** 2018-07-11

**Authors:** S. S. Hakim, M. H. M. Olsson, H. O. Sørensen, N. Bovet, J. Bohr, R. Feidenhans’l, S. L. S. Stipp

**Affiliations:** 1 Nano-Science Center, Department of Chemistry, University of Copenhagen, Universitetsparken 5, 2100 Copenhagen, Denmark; 2 DTU Nanotech, Department of Micro- and Nanotechnology, Technical University of Denmark, Ørsteds Plads, 2800 Kgs, Lyngby, Denmark; 3 Niels Bohr Institute, University of Copenhagen, Blegdamsvej 17, 2100 Copenhagen, Denmark

Correction to: *Scientific Reports* 10.1038/s41598-017-06977-4, published online 08 August 2017

This Article contained an error in the legend of Table 1 where,

“For each interactors, the interacting TANC protein, the detection method and the binding region (experimentally validated) are here listed. Y2H: Yeast two hybrid; Co-IP: Co-immunoprecipitation; SPR: Surface plasmon resonance HTS: High-Throughput System; AC: Affinity Capture; PL: Proximity Label; MS: Mass spectrometry; CLIP: Cross-Linking ImmunoPrecipitation SF-TAP/MS: systematic tandem affinity purifications coupled to mass spectrometry. LIG_PDZ_Class_1: PDZ-binding motif; LIG_EVH1_1: Proline-rich motif binding to signal transduction class I EVH1 domains; DEG_SCF_TRCP1: SCF-betaTrCP1 complex target site; MOD_LATS_1: phosphorylation motif recognised by the LATS kinases; DOC_PP1_RVXF_1: PP1 docking motif; LIG_14-3-3_2: phospho-motif mediating the interaction with 14-3-3 proteins; LIG_Actin_WH2_2: Actin-binding motif; TRG_NES_CRM1_1: Nuclear Export Signal.”

now reads:

“The tabulated parameters are obtained from fitting the reflectivity data with box models. For models with more than one layer, the layer closest to the calcite surface is indexed 1 (e.g. Methanol-1). The density for calcite (2.71 g/cm^3^) and helium (0.03 g/cm^3^) were kept fixed during the fitting procedure, and their thicknesses were infinite. Error estimates were obtained for every parameter by varying the parameter until the χ based R-value is changed by 5% [70, 71]. The method does not reveal correlations between the parameters. On the right side, theoretical bulk density and the molecular length of the molecules are given for comparison.”

